# Motor imagery and action-observation in neurorehabilitation: A study protocol in Parkinson's disease patients

**DOI:** 10.3389/fneur.2022.990618

**Published:** 2022-10-04

**Authors:** Beatrice Rizzi, Christian Nuresi, Claudio Rovacchi, Massimo Bacchini, Federica Savi, Lucia Falco, Luca Schianchi, Augusto Scaglioni, Chiara Ciracì, Cosimo Costantino, Giovanni Buccino

**Affiliations:** ^1^Department of Neuromotor Rehabilitation, Santa Maria ai Servi Center, Don Carlo Gnocchi Foundation ONLUS, Parma, Italy; ^2^Department of Medicine and Surgery, University of Parma, Parma, Italy; ^3^Division of Neuroscience, Università Vita-Salute San Raffaele, Milan, Italy; ^4^IRCCS San Raffaele, Milan, Italy

**Keywords:** action observation, motor imagery, neurorehabilitation, Parkinson's disease, study protocol

## Abstract

**Introduction:**

Action Observation Treatment (AOT) and Motor Imagery (MI) represent very promising cognitive strategies in neuro-rehabilitation. This study aims to compare the effectiveness of the two cognitive strategies, taken alone or combined, in Parkinson's disease patients.

**Material and methods:**

This study is designed as a prospective randomized controlled trial, with four arms. We estimated a sample size of 64 patients (16 in each treatment group) to be able to detect an effect size of *F* = 0.4 with a statistical significance of 0.05. Primary outcomes will be functional gains in the FIM and UPDRS scales. Secondary outcome measure will be functional gain as revealed by kinematic parameters measured at Gait Analysis.

**Discussion:**

The results of this trial will provide insights into the use of AOT and MI, taken alone or combined, in the rehabilitation of Parkinson's disease patients.

**Ethics and dissemination:**

The study protocol was approved by the Ethics Committee of the Don Gnocchi Foundation. The study will be conducted in accordance with the 1996 World Medical Association guidelines and according to good clinical practice. The study has been registered on clinicaltrial.gov under the following code: AOTPRFDG. Dissemination will include both submission of the study to peer-reviewed journals and discussion of the study protocol at conferences.

## Introduction

There is increasing evidence in neuroscience of the existence of a mirror mechanism directly matching observed actions on the neural substrates sub-serving their execution (1, 2). It has been forwarded that this mechanism allows individuals to understand other people's actions on an experiential basis, because of the re-enactment of the motor representations also involved in the actual execution of the observed actions. At neural level this mechanism may be grounded on the existence of mirror neurons first described in monkey pre-motor cortex (3, 4). These neurons discharge during the execution of goal directed actions, as well as during the observation of those same actions or related ones. In more general terms, experimental evidence supports the notion that the neural substrates involved in action execution are recruited whenever actions are re-enacted, like for example in motor imagery or even during dreams with motor content (5). In recent times, it has been proposed an action re-enactment also during the processing of action related language, that is verbs typically describing an action (6, 7). The notion of action re-enactment, based on visual stimuli or on an internal mental rehearse of an action in the absence of overt movements, has recently inspired the use of the action-observation treatment and the motor imagery practice as neurocognitive rehabilitation strategies.

In action observation, patients are shown a video clip representing a goal-directed action (e.g., grasping an object). Subsequently, after observing this video, the patient is asked to repeat the action. So far, treatment with AOT has been shown to be effective in a variety of different pathologies such as ischemic stroke (both chronic and subacute; both on walking and upper limb function) (8–10), cerebral palsy (11–13); Parkinson's disease (14, 15) and also in orthopedic patients with hip fractures (16).

Jeannerod defines motor imagery as the ability to “mentally rehearse simple or complex motor acts that are not accompanied by overt body movements” (17). From a cognitive point of view, it is a perceptive process that occurs in absence of external stimuli. Motor imagery was first studied in cognitive psychology and neuropsychology (18), then applied to sport (19, 20) in order to improve the physical performance of athletes and has recently been applied in rehabilitation medicine to improve the physical recovery of patients with neurological disabilities (21–23). Motor imagery is associated with the activation of several cortical areas that are also involved in action observation and execution (24–27). In particular, the areas that are activated by motor imagery are the ventral premotor cortex (PMv), inferior frontal gyrus (IFG), inferior parietal lobule (IPL), supramarginal gyrus (SMG), sensorimotor area (SMA), cerebellum and basal ganglia (28, 29).

Parkinson's disease is the second most common neurodegenerative disease. In the coming decades, the prevalence of the disease is expected to increase, partly due to the increasing average age of the population. This trend will consequently lead to an increase in the costs related to the medical and social management of the disease, most of which are attributable to the more advanced stages of the disease and directly related to disability, increased care burden and therapeutic complications (30, 31).

Recently, several studies have focused on the applicability of action observation treatment (15, 32) and motor imagery (33, 34) in the rehabilitation of patients with Parkinson's disease. While AOT appears to be effective in improving motor performance, the effectiveness of Motor Imagery is not yet well defined also due to the presence of contrasting data in the literature. This may also be linked to the fact that the subject's ability to imagine depends on many factors (one in particular is the anatomical integrity of the cortical structures connected to this ability), but also to the fact that this ability is poorly investigable (35) and recently a systematic review has emphasized that some areas also involved in Parkinson's disease, if affected, can reduce the capacity to imagine actions (36).

Based on those evidence, we came up with the concept of evaluating the effectiveness of these two cognitive strategies, motor imagery and action observation, in the rehabilitation of Parkinson's disease patients. Based on the ongoing debate and the evidence that action observation is more effective than motor imagery in learning a novel, complex motor task in healthy individuals (37), the first point we want to assess is whether Motor Imagery and action observation are similarly effective, when taken alone, in Parkinson's disease patients. Second, this study aims to assess whether the combination of the two is more effective than the individual treatments. In fact, recent evidence show that motor performance is improved when motor imagery is associated with synchronous action observation of the imagined actions (38).

## Methods and analysis

### Study design

The aim of the study is to evaluate the efficacy of action-observation (AOT) and motor imagery (MI) treatment on the improvement of function and motility in patients with Parkinson's disease with a disease stage II or III according to Hoehn and Yahr. The study was approved by the ethical committee of the IRCCS Don Gnocchi, Milan. The study has been registered to clinicaltrial.gov with the Study ID number: AOTPRFDG. All procedure will be conducted according to the World Medical Association (WMA - 1996) and, when applicable, according to the procedure recommended by the Italian Association of Psychology (AIP). Before starting the study, each patient will be asked for written and verbal informed consent. Motor and functional assessments will be performed at T0 (baseline assessment)–T1 (4 weeks later, at discharge) and T2 (2 months after discharge, as outpatients).

### Study sample and inclusion/exclusion criteria

Patients will be selected from those referred to the intensive rehabilitation unit and Parkinson's service of Don Carlo Gnocchi Foundation, Santa Maria ai Servi Center, Parma. In this setting, patients, aged between 55 and 75 years and with a disease stage II or III according to Hoehn and Yahr, will be enrolled. Exclusion criteria will be the presence of aphasia (evaluated with the token test), hemispatial neglect (assessed with the line bisection test of the Behavioral Inattention Test battery), global cognitive impairment (assessed with the Mini Mental State examination—MMSE, score <24/30), motor apraxia (assessed with the De Renzi gesture imitation test), and major depression (assessed by the Beck Depression Inventory II).

### Randomization

Each patient will then be associated with a randomly generated numerical code and randomly assigned to one of four groups: Motor Imagery + Conventional Treatment; AOT + Conventional Treatment, AOT + Motor Imagery + Conventional Treatment and Control Group + Conventional Treatment. The randomization will be carried out by an independent researcher who will be not involved further in the study.

### Sample size

The sample size has been defined by the achievement of outcome measures. In this study, our outcome measures are an improvement in motor functions and kinematic parameters of patients suffering from Parkinson's disease, as revealed by means of functional scales (FIM, UPDRS) and gait analysis. In order to detect an effect size of *F* = 0.4 (medium to large effect size, according to Cohen, 1988) (39) with 80% power and 0.05 alpha in a mixed subjects design (4 groups and 3 time points), G^*^Power suggests that a total sample size of 64 participants (16 participants for each group) should be enrolled. We plan a sample size of *N* = 80 (*N* for each group = 20) that will be more than adequate to control for a potential participants' drop out. Furthermore, this sample size will be adequate to ensure that data can be interpreted separately, for each group.

### Blinding

Patients in each group will be instructed in the specific task for that group. An experienced, blinded researcher will administer functional scales to enrolled subjects at T0, T1, and T2. Another experienced blinded operator will perform the gait analysis at T0, T1, and T2.

### Treatment protocol

**Figure 1:**
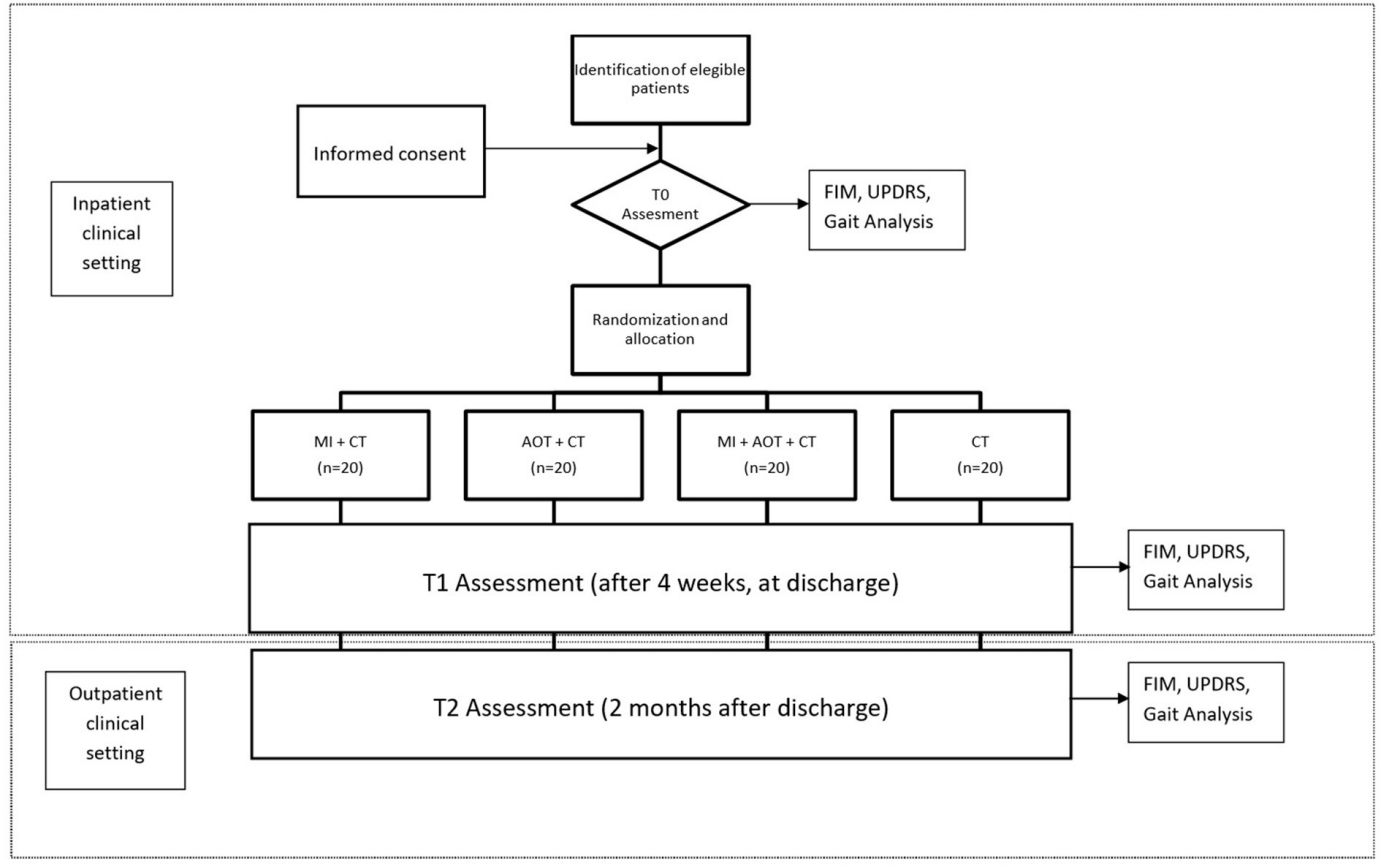
Flow chart of the study protocol.

Enrolled patients will undergo a standard neurological and physiatric evaluation, a neuropsychological assessment, and gait analysis. Function and independence will be quantified with the FIM scale and UPDRS. In addition, a visual and kinesthetic imagination scale (MIQ-R) will be delivered to each patient enrolled. The patients then will undergo a 4-week inpatient treatment (excluding weekends), at the end of which the functional assessment (FIM scale and UPRDS) as well Gait Analysis will be repeated. The euro Quality of Live (eQoL) questionnaire will be administered to each subject on admission and at discharge. During hospitalization, at a fixed day time, the patient will undergo a conventional rehabilitation treatment administered by a physiotherapist different from the one involved in the study. All treatments will be carried out in ON phase patients. Study protocol is summarized in Figure 1. In the follow-up evaluation at T2 participants will be assessed as outpatients.

### Conventional treatment

Conventional treatment will consist of 10 min of active kinesis with limb and trunk mobilization exercises; 5 min of ischio-crural, adductor, hip flexor and plantar flexor foot muscles stretching; 10 min of postural spine extension exercises; 5 min of gait training to improve gait phases and dynamic balance and 5 min of coordination exercises.

### Action-observation treatment

Patient will undergo AOT according to the procedure described in a comprehensive review in the field (40). During a typical AOT rehabilitation session, patients train one single daily life action. Actions to be trained are chosen among those most frequently carried out in everyday life. An AOT rehabilitation session consists of an observation phase and an execution phase. During the observation phase, the patient sits in front of a computer screen and has to observe a videoclip, showing the execution of a specific action. The presented action can be divided into four motor acts, each lasting 3 min, for a total of 12 min. Motor acts are defined as the different action segments into which the action can be divided. For example, washing one's hands can be divided into the following motor acts: (I) applying soap on wet hands; (II) scrubbing hands for a few seconds; (III) rinsing hands under running water; (IV) drying them with a towel. Each motor act is performed by both an actor and an actress and is presented from different perspectives in order to catch the attention of the patient. After observing a single motor act, the patient moves to the action execution phase where he/she is required to perform the same action, at the best of his/her abilities, for 2 min. Every object present in the video clip is provided at hand to the patient during this phase. Indeed, it is well known that objects can recruit the motor representation of an action, thus further enhancing the re-organization of the motor system (41–43).

### Motor imagery

The proposed motor imagery activity will be carried out by showing the same videos used in AOT. In this group each video will be shown to the patient just for a few seconds. Following this short observation period, the patient will be asked to imagine himself/herself performing the observed action for 3 min (the same time devoted to observation in the AOT group). Following the imagery task the patient will be asked to reproduce the imagined action for 2 min as patient in the AOT group. Every now and then, the physiotherapist will monitor the imagery activity by asking the patient at what time point of the action he/she is.

### Action observation treatment + motor imagery

This group will undergo AOT as already described. After observing each motor act, patients in this group will be asked to imagine the observed model for 3 min in first person perspective (as if he/she should be performing the seen motor act). At the end of the motor imagery, patients in this group will be asked to perform the observed and imagined motor sequence at the best of their ability for 2 min. As usual, the physiotherapist will help patients to pay attention to the task and sustain their motivation.

### Control treatment

For the control group, twenty videos will be prepared representing situations with no specific motor content. Also in this case videos will consist of 43-min parts with a total duration of 12 min. The patient will be asked to watch the videos. After observing this motorically neutral videoclips, the patients will be requested verbally to perform one of the motor activities actually executed by patients enrolled in the AOT or MI groups. In other words, the execution phase in this group will overlap that of patients in the other study groups. In this manner patients in this group will spend the same amount of time in the observation phase, as well as in the execution phase, as patients in the study groups.

### Pre-clinical and clinical evaluation

The Clinical evaluation of patients will take place in the intensive rehabilitation department of the Don Carlo Gnocchi Foundation, Santa Maria ai Servi Center, Parma.

### Demographic and clinical characteristics

The patients will be classified according to Hoehn and Yahr scale. This classification allows the degree of disability to be assessed on a scale of I to V. Patients classified from stage I to stage III are defined as mildly disabled, while those in stage IV or V are defined as severely disabled (44). The HY scale is widely used in clinical settings to classify the level of disability of Parkinson's patients because it is simple, easily applicable and reliable. It also has a good correlation with the UPDRS scale (45). The anagraphic (age and sex) and clinical characteristics of the subjects will be assessed at T0 (baseline) in all groups to evaluate their homogeneity.

### Cognitive and neuropsychological assessment tools

#### Token test

Token test is the most widely used clinical tool to assess the presence of aphasia. It consists of 36 items in which the patient is required to understand orders of increasing complexity. It has a good test-retest reliability (46) and intra-rater reliability while the inter-rater one is good although not in all its parts (47).

#### Line bisection test of BIT battery

The Line bisection test allows to assess the presence of neglect. It is performed by presenting the partecipant with a series of horizontal lines and asking him/her to bisect them in the center. If the patient has neglect, he/she will fail to dissect them in the midline. Together with other tests, line bisection is included in the Behavioral Inattention Test (48). It is an easy test to administer and does not require any specific materials.

### Mini-mental state examination

The MMSE test is a practical, well spread, questionnaire including 11 items that aims to evaluate cognitive functions. It has a high sensitivity, a moderate-to-high level of reliability, validity and excellent reliability at test-retest. Indeed it is not completely reliable in assessing mild cognitive impairments (49, 50).

### De Renzi imitation gesture test

It is a test that can be easily administered and that allows with a certain sensitivity to recognize if the patient has ideomotor apraxia (51).

### Beck depression inventory II

The BDI I-II is a valuable tool to detect if the subject is suffering from depression. It is a self-evaluation test and has good internal validity and good test-retest reliability (52).

### Outcome measures

Primary outcome measures will be score changes in two functional scales: FIM and UPDRS. Secondary outcome measures will be changes in kinematic parameters as revealed by gait analysis.

### Unified Parkinson's disease rating scale (UPDRS)

The scale is divided into four macro-areas: 1. mental activities, behavior and mood; 2. activities of daily living; 3. motor assessment; and 4. complications. One of the main advantages of the UPDRS scale is that it provides a global assessment of the patient with Parkinson's disease, including both motor and non-motor aspects and any complications related to treatment. Other advantages of using this scale are its ease of use and speed of administration (53). It has also demonstrated excellent internal reliability (54), inter-rater validity (55), and good test-retest validity (56). From previous studies it seems that AOT has a significant impact on the functional recovery of PD patients (15).

### Functional independence measure

The FIM scale is widely used in rehabilitation settings to assess the level of assistance a patient needs in performing daily living activities. It includes 18 items with scores ranging from 1 to 7. The items are grouped into six areas, each with a specific competence: 1. self care; 2. sphincter control; 3. mobility; 4. locomotion; 5. communication; 6. social cognition. The maximum score is 126 and represents the total independence of the subject. The minimum score is 18 and represents the subject's total dependence in all activities of daily living (57, 58). It has good inter-rater reliability, although trained personnel are needed to administer it properly (59).

### Gait analysis

In addition to the two above-mentioned outcomes, in our study we will use gait analysis to assess kinematic parameters following the treatment. The Optoelectronic Motion Analysis System EL.I.TE (BTS, Milan, Italy) will be used for this evaluation (60–62).

### Data management

To ensure the anonymity of the participants enrolled in the study, each patient will be assigned an identification code. A list of identification codes will be then generated and stored separately. All descriptive data will be collected and stored on data files in protected computer networks.

### Statistical analysis

The statistical analysis of the data will be carried out by a statistician using the R software.

Given the design of the protocol (4 Groups X 3 Time-points), a mixed between-within ANOVA model with Greenhouse-Geisser correction will be used as a statistical test for this purpose. Statistical significance will be set at 0.05 to reject the null hypothesis.

## Discussion

AOT and MI are two cognitive strategies useful in neuro-rehabilitation. In the literature, there are only preliminary evidence in favor of their effectiveness in the motor recovery of patients with Parkinson's disease. However, there is still little evidence as to which of the two strategies is best for this purpose or whether, combining the two, the benefits for patients can be maximized. In healthy participants by comparing these two strategies, it has been found that Action-Observation is more effective than MI in improving the learning of complex novel motor tasks (37). This greater effectiveness may derive from various factors, one of which may be linked to the method itself. While during observation the motor task shown is contextualized and performed “correctly”, in motor imagery the effectiveness depends, in part, also on the participant's ability to re-enact that specific action in a proper manner. When applying these strategies in neuro-rehabilitation it's worth stressing that during action observation, at difference with motor imagery, the clinician has greater control over the attention, motivation and correct execution of the task by the participant throughout the treatment. We expect that this study will bring further evidence in understanding which of the two strategies is more effective in the rehabilitation of Parkinson's Disease patients and whether combination of the two methods may be more effective than the single ones.

Another aspect we would like to address concerns the effectiveness of these cognitive strategies at long term follow up as compared to conventional rehabilitation treatment. We know that rehabilitation treatment is certainly beneficial in this type of patients, but this benefit is often described in the literature as short-termed (63). The possibility of pursuing rehabilitation in a different setting from a conventional one (e.g., at hospital and/or in a rehabilitation center) is therefore of primary importance to ensure and maintain good functionality and this represents a strength of cognitive strategies, like AOT and MI, since they can be easily repeated over time and applied in different contexts. Indeed, there is some preliminary evidence for the efficacy of AOT in telerehabilitation (64, 65). Moreover, by means of AOT and possibly of motor imagery, all actions can be trained and during the training observed and imagined actions can be tailored to the needs of patients to improve the functionality and motility of any body district (upper limbs, lower limbs, trunk and mouth) (40).

In recent years, the concept of Health Literacy (HL) has developed greatly. HL is defined by the WHO as “The cognitive and social skills which determine the motivation and ability of individuals to gain access to understand and use information in ways which promote and maintain good health” (66, 67) and represents a fundamental theme on which different health care models around the world are focusing to cope with an increasing demand for access to care by the world's population. We believe that cognitive strategies like AOT and motor imagery fit perfectly into this framework, representing a powerful treatment tool in the hands of the patient, making him/her an actor of his health with considerable benefit for himself/herself and cost reduction for the global health system.

Lastly, we hope that this work will stimulate debate on this topic, enabling the development of increasingly specific protocols tailored to the patient's functional needs.

## Ethics statement

The studies involving human participants were reviewed and approved by IRCCS Don Gnocchi, Milan. The patients/participants provided their written informed consent to participate in this study.

## Author contributions

GB conceived the study and revised the manuscript. BR, CN, MB, CR, FS, LS, LF, AS, CCi, and CCo wrote specific parts of the first draft of the manuscript and revised it. All authors contributed to the article and approved the submitted version.

## Funding

This study was funded by the Cariparma Foundation, Parma.

## Conflict of interest

The authors declare that the research was conducted in the absence of any commercial or financial relationships that could be construed as a potential conflict of interest.

## Publisher's note

All claims expressed in this article are solely those of the authors and do not necessarily represent those of their affiliated organizations, or those of the publisher, the editors and the reviewers. Any product that may be evaluated in this article, or claim that may be made by its manufacturer, is not guaranteed or endorsed by the publisher.
